# The impact of perception of discrimination and sense of belonging on the loneliness of the children of Chinese migrant workers: a structural equation modeling analysis

**DOI:** 10.1186/1752-4458-8-52

**Published:** 2014-12-12

**Authors:** Dongyang Liu, Xiaobo Yu, Yuncai Wang, Haiqin Zhang, Guofang Ren

**Affiliations:** Institute of Teacher Education, Shanxi Normal University, Linfen, China; School of Education, Anyang Normal University, Anyang, China

**Keywords:** Perception of discrimination, Sense of belonging, Loneliness, Children of migrant workers

## Abstract

**Background:**

The children of migrant workers can experience several mental health problems after they enter the cities, among which, loneliness is the most prominent and the most common psychological problem. The current study aimed to examine the impact of discrimination perception on loneliness of migrant children, mainly focused on confirmation of the mediator role of sense of belonging.

**Method:**

357 children of Chinese migrant workers were as participants involving in this research. Data were collected by using the Perception of Discrimination Scale, Sense of Belonging Instrument, and the Social and Emotional Loneliness Scale.

**Results:**

The results revealed that both perception of discrimination and sense of belonging were significantly correlated with loneliness of the children of migrant workers. Structural equation modeling indicated that sense of belonging partially mediated perception of discrimination to job loneliness.

**Conclusions:**

Sense of belonging played a significant role in the relation between perception of discrimination and loneliness of the children of migrant workers.

## Introduction

More surplus rural laborers pour into the cities with the rapid development in Chinese society and economy, which form the greatest population migration in the world [1]. These peasants leave the countryside to work in cities are called rural migrant workers (migrant workers for short). Large amount of second generation immigrants have appeared in the cities with the migration of rural laborers with their families [2]. However, these school-age children who follow their parents cannot enjoy the equal rights of education as local kids because of dual economic and social system based on census register and urban and rural division [3]. These children become a special vulnerable group in society [4]. According to the data published by the Chinese Ministry of Education in 2012, a total number of 43 million children of migrant workers should accept compulsory education [5]. The children of migrant workers in China are similar to the migrant children or mobile children in Western countries. According to previous research, immigration and its process can influence mental health [6, 7]. Being children of migrant workers, they are faced with the adjustments and challenges in family, life, and study. Their psychological health conditions are greatly threatened [4] by internal and external factors.

The children of migrant workers can experience several mental health problems after they enter the cities, among which, loneliness is the most prominent and the most common psychological problem [8, 9]. Loneliness is a serious painful state of an individual who feels deficient in quantity and quality [10–12]. According to the study of Wong and colleges, loneliness among children of migrant workers is more prominent than that in ordinary children, and most of the children of migrant workers experience strong sense of loneliness [4]. According to a research on cultural immigration, cross-cultural flow leads to a change on the life course of an individual; therefore, he or she can be stuck in loneliness [13]. Anyone can experience loneliness under the environment of cross-culture; however, the children and youth have higher risks of facing loneliness [14]. In addition, loneliness can lead to other psychological problems. According to research, loneliness causes great pain to children; children under this condition have no sense of belongingness and have low self-respect [15]. In addition, loneliness is related to depression, alcohol abuse, and suicidal behavior [16, 17]. Above all, loneliness has been the most prominent and most serious of all the psychological problems experienced by the children of migrant workers that attracted the attention of some researchers. Several researchers have explored the reasons for the occurrence and development of loneliness among children of migrant workers. The results reveal the following influencing factors: perception of discrimination, social support, sense of belonging, fellow relations, and relationship with parents [4, 9, 18].

Perception of discrimination is a subjective experience; for instance, an individual recognizes unequal treatment because of his membership (including race and household status) in a group [19, 20]. Most migrant workers devote themselves to dirty and hard work. These workers, including their children, live together in the corner and on the edge of the urban city where discrimination and inequity is high. Discrimination against children of migrant workers has gradually attracted the attention of the researchers. An investigation has been conducted to examine whether these children complained to their parents. The result reveals that 24.7% of the children have complained of being discriminated against by city people, whereas 29.3% are worried that children in cities will discriminate them. Children enrolled in the school for migrant workers children may worry more that they are discriminated by others [21], which indicates that these children have obviously felt the low social status and unequal treatment of their parents. According to the research conducted by Li and his colleges, 75.5% of these children reported to have been discriminated [22]. Therefore, the children of migrant workers are facing different kinds of “social exclusion” in school and in society. Such feeling should be guided accordingly to keep them from developing resistance against society, which is harmful to their growth [4].

Belongingness refers to the internal relationship of an individual and the group where he belongs. For instance, an individual recognizes and maintains a certain group [23]. Sense of belongingness is the psychological reflection of such recognition and maintenance [24]. When people experience a sense of belongingness, they tend to be healthy and happy. By contrast, lack of a sense of belongingness will result in negative emotional experience such as anxiety, depression, anger, sadness, and loneliness [25, 26]. Several scholars have demonstrated that perception of discrimination is a key influencing factor on low sense of belongingness [27–29]. To conclude, loneliness is the main psychological problem faced by the children of migrant workers, whereas high perception of discrimination and low sense of belonging are the important influencing factors of loneliness. By using a questionnaire survey, the current study explores and discusses the effect of perception of discrimination and sense of belongingness on the loneliness of the children of the migrant workers.

## Methods

### Participants and procedure

Participants were 357 students from two migrants’ schools in China, and 164 were female and 193 were male. The ages of participants ranged from 12 to 14, with a mean of 12.82 (SD = 0.41). Participants completed the questionnaires in a classroom environment, and received ¥20 as compensation. 357 valid scales were distributed and collected. All participants provided their written informed consent before completing the measures.

### Instruments

#### Perception of discrimination scale

Perceived discrimination Scale of Chinese migrant children, developed by lin, et al., is a 9-item measure measuring the experience of transracial adoption discrimination, including two dimensions, namely, social discrimination and institutionalized discrimination [30]. Some examples of the scale include “I have been excluded or rejected by others because of my parents’ work”, “I feel I am not welcomed in this city”. Ratings were completed on a four-point scale ranging from 1 (strongly disagree) to 4 (strongly agree). This scale is reported to have satisfied reliability and validity [4, 31]. In the present study, the Cronbach alpha coefficients for social discrimination scale and institutionalized discrimination scale were 0.813 and 0.764 respectively.

#### Sense of belonging instrument (SOBI)

The Sense of Belonging Instrument (SOBI), a 20-item measure, consists of two subscales, namely fit and valued involvement. Some examples of the scale include “If I died tomorrow, very few people would come to my funeral”, and “I often wonder if there is anyplace on earth where I really fit in”. Ratings were completed on a four-point scale ranging from 1 (strongly disagree) to 4 (strongly agree). Scale scores are the sum of items with reverse coding of relevant items. Cronbach’s coefficients alpha have been reported to range from .91 to .93 for SOBI [32]. In this study, Cronbach alpha coefficients for fit and valued involvement scales were 0.778 and 0.830.

#### Social and emotional loneliness scale

The Social and Emotional Loneliness Scale was developed by Wittenberg. It consists of 10 items designed to assess social loneliness (SL) and emotional loneliness (EL). Responses to each item are given on a five-point Likert-type scale ranging from 1 (never) to 5 (very often). It includes items such as, “1 have a really nice set of friends”; “I have friends and acquaintances with who I like to be together”; “I feel lonely even when I am with other people”. Scale scores are the sum of items with reverse coding of relevant items [33]. In the current study, the Cronbach alpha coefficient for social and emotional loneliness sub-scale were 0.815 and 0.847.

## Results

### Descriptive statistics and correlation analysis

Means, standard deviations, and intercorrelations for all the variables were presented in Table 1. The results showed that perception of discrimination was negatively correlated with sense of belonging (r = −0.51, p < 0.01), and positively correlated with loneliness (r = 0.47, p < 0.01). In addition, sense of belonging was also negatively correlated with loneliness (r = −0.52, p < 0.01).

**Table 1:** **Means, standard deviations, and correlations of the variables of interest**

	Mean	SD	1	2	3
1. Perception of discrimination	21.66	4.64	1		
2. Sense of belonging	41.96	9.03	−0.51^**^	1	
3. Loneliness	31.81	6.77	0. 47^**^	−0.52^**^	1

Note: ^**^, p < 0.01.

### Measurement model

Confirmatory factor analysis (CFA) was adopted to assess whether the measurement model fit the sample data adequately or not. The following four indices were utilized to evaluate the goodness of fit of the model: (a) Chi-square statistic (χ^2^), χ^2^/df, (b) the Standardized Root Mean Square Residual (SRMR), (c) the Root Mean Square Error of Approximation (RMSEA), and (d) the Comparative Fit Index (CFI) [34, 35]. In this study, a model was considered to have a good fit if all the path coefficients were significant at the level of 0.05, χ^2^/df was below 5, SRMR was below 0.08, RMSEA was below 0.08, and CFI was 0.95 or more.The fully measurement model included three latent constructs (perception of discrimination, sense of belonging and loneliness) and 6 observed variables. The initial test of the measurement model came into being a satisfactory fit to the data: χ^2^/df = 1.47, RMSEA = 0.039, SRMR = 0.012, and CFI = 0.98. All the factor loadings for the indicators on the latent variables were significant (p < 0.001), indicating that all the latent constructs were well represented by their indicators.

### Structural model

**Figure 1:**
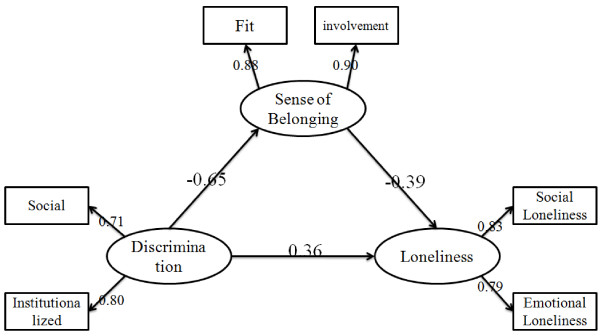
**The mediating role of sense of belonging between discrimination and loneliness.** Note: Factor loadings are standardized (p < 0.05).

Then SEM was used to analyses the mediation effect. First of all, the direct effect of perception of discrimination on loneliness without mediators was tested. The directly standardized path (β = 0.62, p < 0.001) was significantly. Then, a partially-mediated model which contained mediator (sense of belonging) and a direct path from perception of discrimination to loneliness was tested. The results showed that the model goodness of fit showed a very good fit to the data: χ^2^/df = 1.44, RMSEA = 0.035, SRMR = 0.015, and CFI = 0.99. The final model was shown in Figure 1. Taken together, these results showed that both perception of discrimination and sense of belonging have significant impacts on loneliness.

### The confidence interval of direct and indirect effects

The mediating effects of sense of belonging between perception of discrimination and loneliness was tested for a significance by adopted the Bootstrap estimation procedure in AMOS (a bootstrap sample of 1500 was specified). The reason for not using Sobel test, the commonly employed method for examining the statistical significance of a mediation effect, which involves computing the ratio of products of direct effects to their estimated standard error, is that Sobel test requires the products of direct effects follow a normal distribution which is always not accordance with the fact, thus resulted in the reduction of statistical efficacy. However, the bootstrap test actually relies on the 95% confidence intervals from the empirical distribution of indirect effect estimates and Mackinnon suggested that the bootstrap method yields the most accurate confidence intervals for indirect effects [36, 37]. Table 2 shows the indirect effects and their associated 95% confidence intervals. As shown in Table 2, the indirect effect of perception of discrimination on loneliness through sense of belonging was significant. The effect of perception of discrimination on loneliness through sense of belonging was 41.32%.

**Table 2:** **Direct and indirect effects and 95% confidence intervals for the final model**

Model pathways	Estimated effect	95% CI lower bonds	95% CI up bonds
Direct effect			
Perception of discrimination→Loneliness	0.360	0.120	0.670
Perception of discrimination→Sense of belonging	−0.645	−0.784	−0.501
Sense of belonging→Loneliness	−0.387	−0.601	−0.109
Indirect effect			
Perception of discrimination→Sense of belonging→Loneliness	0.250	0.131	0.614

## Discussion

A considerable number of researches have demonstrated that perception of discrimination can lead to negative psychological development [38, 39]. However, the mechanism of relationship between discrimination and psychological health has not yet been solved. Based on the existing theories and empirical research, the effect of perception of discrimination on loneliness (in which sense of belonging plays a mediating role) has been introduced and verified.

According to the analysis of discrimination perception data for the children of migrant workers, these children have experienced discrimination in the cities because their parents are from other places [40]. They feel the unequal treatment from their partners and inside the school. Perception of discrimination is the key influence on loneliness, which is in accordance with the previous research results [4, 9, 18]. When an individual feels discrimination from his or her partner or from the society, he or she will be hesitant to communicate with others and will develop a sense of loneliness [41]. According to the scar theory, negative events can strengthen the passive cognitive style of an individual [42]. Discrimination can develop more negative and sensitive awareness in the environment of the children of migrant workers. Such cognitive style may further strengthen the perception of discrimination, which lead to deeper loneliness [43, 44].

The current research mainly verifies the mediating role of sense of belonging in the relationship between perception of discrimination and loneliness. Based on the social identity theory, social acceptance is a key factor that influences individual psychological health [45]. The awareness of exclusive behaviors come from others; thus, the perception of discrimination may directly reduce the individual sense of being accepted, which causes negative effects to psychological health [46, 47]. First, the children of migrant workers will feel the discrimination of urban children of the same age. Compared with local students, the children of migrant workers have poor economic condition. They live far away from their hometown, and their social network in cities is not yet established. They live in the city, but they do not belong there. They are far away from the rural area, but they are not completely countrymen any more [48]. They have become people who are living at the margin between cities and villages. With the increased perception of discrimination, the children of migrant workers have a stronger feeling unacceptance from their schoolmates. These children are also encountering discrimination from the social system. For example, they cannot completely participate in the school entrance examination in flow-in cities [49]. Thus, they return to their hometowns for the entrance examination. These systematic limitations have an effect on the sense of belongingness of children in schools or cities, which lowers their sense of belongingness in the city. On the one hand, sense of belonging stimulates the feeling of being accepted. On the other hand, it can improve the self-respect and self-esteem of an individual [50]. These active emotions become the protective factors of psychological health, which reduce sense of loneliness. According to the hierarchical theory of needs, people need sense of belongingness and love. The lack of sense of belongingness can cause harmful effects on the mental health of an individual [51].

The current result indicates that the promotion of the psychological health of the children of the migrant workers requires urban students to be educated and to treat them kindly. The discrimination policies on these children should be eliminated. In turn, the children of migrant workers should have stronger identification in cities and schools. They should feel they are one of the students in the school and of the city and accept the equality between them and the people living in the cities.
